# Polyisocyanide Quaternary Ammonium Salts with Exceptionally Star-Shaped Structure for Enhanced Antibacterial Properties

**DOI:** 10.3390/polym14091737

**Published:** 2022-04-24

**Authors:** Hongguang Zhang, Lijia Liu, Peng Hou, Hong Pan, Shuang Fu

**Affiliations:** 1 College of Pharmacy, Qiqihar Medical University, Qiqihar 161006, China; zhanghg@qmu.edu.cn (H.Z.); houp@qmu.edu.cn (P.H.); panh@qmu.edu.cn (H.P.); 2 Key Laboratory of Superlight Materials & Surface Technology, Ministry of Education, Institute of Advanced Marine Materials, College of Materials Science and Chemical Engineering, Harbin Engineering University, Harbin 150001, China; liulijia@hrbeu.edu.cn

**Keywords:** star-shaped structure, polyisocyanide, quaternary ammonium salt, antibacterial

## Abstract

The development of non-polluting and non-hazardous polymeric antimicrobial agents has become a hot issue in current research and development. Among them, polymer quaternary ammonium salts are thought to be one of the most promising materials for antibacterial efficacy. Here, we present an efficient strategy for synthesizing polyisocyanide quaternary ammonium salts (PQASs) with a novel star-shaped structure. Benefitting from the novel structure, increased cation density and enhanced water solubility, the prepared star polyisocyanide quaternary ammonium salts (S-PQASs) exhibit excellent antibacterial properties against *Escherichia coli* (*E. coli*) and *Staphylococcus aureus* (*S. aureus*). In particular, S-POcQAS-M_50_ (where M stands for isonitrile monomer and 50 stands for the initial feeding ratio) showed the best antimicrobial activity with minimum inhibitory concentration (MIC) of 17 and 20 µg/mL against *E. coli* and *S. aureus*, respectively. It was also found that the unique star-shaped structure can give QASs with improved antimicrobial performance compared with our previously prepared linear quaternary ammonium salts (L-PQASs). These results demonstrated that the antibacterial activity of QASs is closely related to its structure. This work provides an idea for the design of efficient polymeric antimicrobial agents.

## 1. Introduction

Due to the irrational use of antibiotic drugs, the increase in drug-resistant bacteria and viruses has led to an increasingly hostile environment for human survival [1,2,3,4]. The research and development of organic polymeric antibacterial compounds are recognized as one of the most effective strategies to alleviate the environmental issues caused by the increasing abuse of antibiotics [5,6,7,8], which is ascribed to its excellent processing properties, good stability and non-volatility [9,10,11]. Therefore, the design of an organic polymer with excellent antimicrobial properties using simple methods has received interest in the research community.

In the past decades, various organic polymeric antibacterial agents have been developed, such as polymer quaternary ammonium salts (PQASs) [12,13,14], polymer quaternary phosphonium salts (PQPSs) [15,16], chitosan [17,18,19,20], polymer guanidine [21], etc. Among them, PQASs has attracted great attention owing to its fast sterilization performance, excellent structure stability, and low cost [22,23,24]. To date, numerous studies have reported that various factors, including the structure and molecular weight of the polymer, structure of quaternary ammonium groups, water-solubility and biocompatibility of the polymer, can significantly affect the antimicrobial activity of PQASs. For example, Zhang et al. [25] have developed six β-pinene quaternary ammonium salts and found that compound **4a** showed the best antibacterial action, suggesting that the antibacterial activity of quaternary ammonium salts is closely related to their structure. Dong et al. synthesized phosphonate/quaternary ammonium copolymers exhibiting remarkable antibacterial activity, and they found that the bactericidal ability of the copolymer increased with the increase in cationic content in the copolymer [26]. Brittany et al. found that the bactericidal activity of quaternary ammonium-modified poly(amidoamine) dendrimers was dependent on dendrimer generation [27]. However, despite the considerable endeavors that have been devoted, the current PQASs still have the following several limitations: (1) the poor stability of monomers; (2) the low quaternization of PQASs; and (3) the poor water-solubility of PQASs. Therefore, exploring novel PQASs that can overcome the above disadvantages is of great importance.

In our previous work, a series of L-PQASs have been reported for highly efficient antibacterial and antitumor applications [28]. In recent years, branched polymers with star-shaped, dendritic and hyperdendritic structures have attracted attention as potential antimicrobial agents. Many studies in the literature have reported that the antimicrobial activity of the dendrimers and the hyperbranched quaternary ammonium compounds was higher than the linear polymers [29,30]. For example, Lia A. T. W. Asri et al. found that hyperbranched Si-HB-PEI^+^ coatings exhibited high contact-killing efficacies against *S. aureus*. Furthermore, the hyperbranched Si-HB-PEI^+^ coatings (10 wt %) did not negatively affect the adhesion and proliferation of fibroblasts [31]. Yudan Wang et al. developed a novel crosslinked hyperbranched cationic polymer membranes (cHCPMs) and found that the membranes showed excellent broad-spectrum antibacterial and antibacterial adhesion properties against *E. coli* and *S. aureus* [32]. Chen et al. functionalized poly(propylene imine) and poly(amidoamine) dendrimers with dimethyl dodecyl ammonium groups and found that dendrimers macromolecules containing 16 QAS groups exhibited two orders of magnitude higher bactericidal efficiency against Gram-negative bacteria than those with mono-functional counterparts [33,34]. It is well known that polymers with dendritic structures possess many unique properties, such as high solubility, low viscosity, and large numbers of functional groups [35]. Hence, the development of hyperbranched quaternary ammonium salt antibacterial agents has become a hot focus of research.

In this work, we further optimized the experimental conditions and successfully synthesized S-PQASs with novel star-shaped hyperbranched structures by using polyisocyanide as the backbone. Due to the sequential insertion of isonitrile groups in the polyisocyanide backbone, a tightly structured carbon chain is formed in the polymer with a small spacing between the side chains, which enables effective synergistic effects between the functional groups on the side chains. Notably, the prepared S-POcQASs exhibited advanced antibacterial activity against both *E. coli* and *S. aureus* in comparison with our previously prepared L-POcQASs. The results of the experimental demonstrate that the improvement of antibacterial activity is mainly ascribed to the optimization of the structure of PQASs.

## 2. Materials and Methods

### 2.1. Materials

Tetrakis(triphenylphosphine)palladium(0) (Pd(PEt_3_)_4_) (CAS: 14221-01-3), copper iodide (CuI) (CAS: 7681-65-4), 1,3,5-tribromobenzene (C_6_H_3_Br_3_) (CAS: 626-39-1), triphenylphosphine (PPh_3_) (CAS: 603-35-0), (trimethylsilyl)acetylene (C_5_H_10_Si) (CAS: 1066-54-2), dichloromethane (DCM) (CAS: 75-09-2), anhydrous magnesium sulfate (MgSO_4_) (CAS: 139939-75-6), sodium bicarbonate (NaHCO_3_) (CAS: 144-55-8), petroleum ether (PE) (CAS: 8032-32-4), ethyl acetate (EA) (CAS: 141-78-6), tetrabutylammonium fluoride (TBAF), solution in THF (1M) (CAS: 429-41-4), N-hexane (C_6_H_14_) (CAS: 110-54-3), dichlorobis(triethylphosphine)-palladium(II) (Pd(PEt_3_)_2_Cl_2_) (CAS: 28425-04-9), cuprous chloride (CuCl) (CAS: 7758-89-6), triethylamine (NEt_3_) (CAS: 121-44-8), and *N*,*N*-dimethyloctylamine (C_10_H_23_N) (CAS: 7378-99-6) were purchased from Sigma-Aldrich (St. Louis, MO, USA). All other raw materials used for the preparation of monomers and S-POcQASs were the same as those used for our previous preparation of L-POcQASs. *E. coli* (ATCC25922) and *S. aureus* (ATCC25923) were obtained from the first affiliated hospital of Harbin Medical University (Harbin, China).

### 2.2. Synthesis of Palladium Catalyst with Star-Shaped Structure

**Scheme 1:**
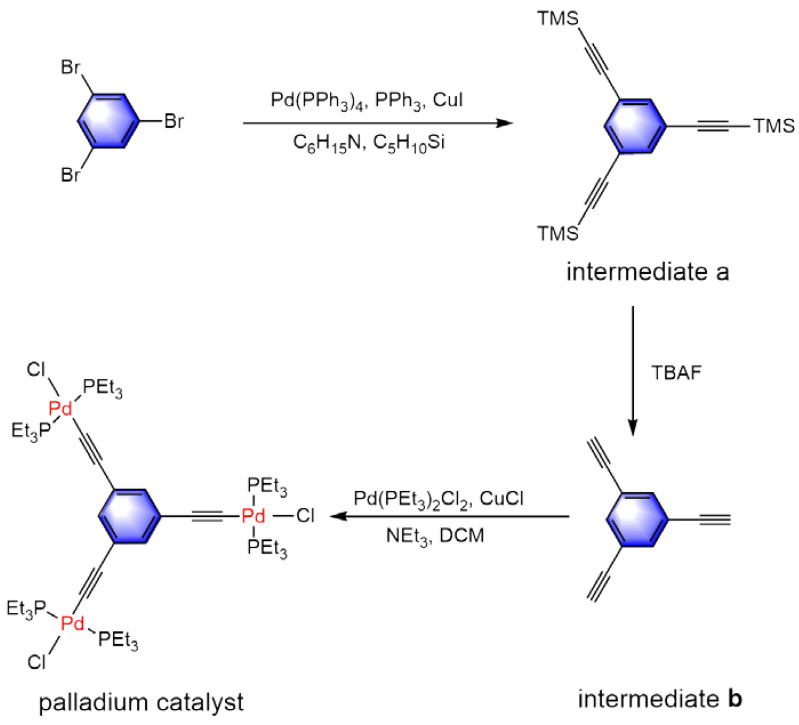
Chemical structure and synthetic procedures of palladium catalyst.

The synthesis of a palladium catalyst with a star-shaped structure was carried out, and the reaction process is shown in Scheme 1.

#### 2.2.1. Synthesis of Intermediate **a**

The synthesis of intermediate **a** was conducted via a classical Sonogashria coupling reaction. First, 3.14 g (10.01 mmol) of 1,3,5-tribromobenzene, 0.69 g (0.60 mmol) of Pd(PEt_3_)_4_, 0.16 g (0.60 mmol) of PPh_3_, and 0.12 g (0.60 mmol) of CuI were added to a 100 mL two-necked flask containing 50 mL of triethylamine. Afterward, 5.1 mL (36.10 mmol) of (trimethylsilyl)acetylene was added to the reactor under N_2_ conditions, and the reaction was continued for 12 h under constant stirring. When the substrate was completely consumed using TLC analysis, the mixture was cooled to room temperature, and the filtered liquid was spin-dried under reduced pressure. The solid was washed with distilled water (2 × 30 mL) and saturated NaHCO_3_ solution (2 × 30 mL). Subsequently, it was dried over anhydrous MgSO_4_, filtered, and the filtered liquid was spin-dried under reduced pressure. Finally, the pale yellow intermediate **a** (2.09 g, yield 57.2%) was obtained by column chromatography (silica gel, PE:EA = 10:1 *v*/*v*) for further purification.

#### 2.2.2. Synthesis of Intermediate **b**

First of all, the above-prepared intermediate **a** (1.83 g, 5.00 mmol) was dissolved in a two-necked flask containing dry THF (30 mL) under N_2_ conditions. Then, 5 mL of THF solution of tetrabutylammonium fluoride (TBAF) (1M) was added dropwise to the above solution under ice water bath conditions. After continuous stirring for 3 h at room temperature, the intermediate **a** was judged to be completely consumed by TLC analysis. Subsequently, the reaction was quenched by the immediate addition of deionized water. The organic phase was washed with deionized water, and the solvent was removed in a rotary evaporator to obtain the crude product. Finally, the crude product was recrystallized by N-hexane to obtain a needle-shaped crystal of intermediate **b** (0.47 g, yield 62.8%).

#### 2.2.3. Synthesis of Palladium Catalyst

The palladium catalyst with a star-shaped structure was prepared according to the literature [36]. In detail, 0.08 g (0.55 mmol) of the above-prepared intermediate **b**, 0.75 g (1.80 mmol) of Pd(PEt_3_)_2_Cl_2_, and trace CuCl were added to a 50 mL two-neck flask. Then, 25.0 mL of CH_2_Cl_2_ and 5.0 mL of NEt_3_ were mixed into the solution under dark and N_2_ condition. After continuous stirring for 8 h, the intermediate **b** was judged to be completely consumed by TLC analysis. Subsequently, the solvent was removed, and column chromatography (silica gel, PE:EA = 7:1 *v*/*v*) was used to purify the solid. Finally, the crude product was recrystallized from DCM/N-hexane as a white crystal (0.24 g, yield 37.1%).

### 2.3. Synthesis of Isonitrile Monomer

**Scheme 2:**
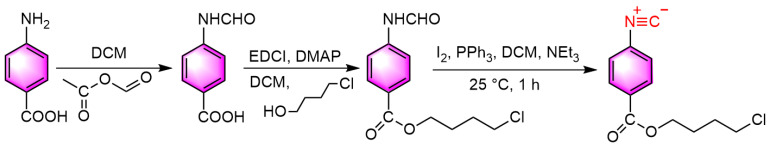
Chemical structure and synthetic procedures of isonitrile monomer.

The synthesis procedure and detailed characterization of the isonitrile monomer was reported in our previously published work, as shown in Scheme 2.

### 2.4. Synthesis of S-POcQASs

**Scheme 3:**
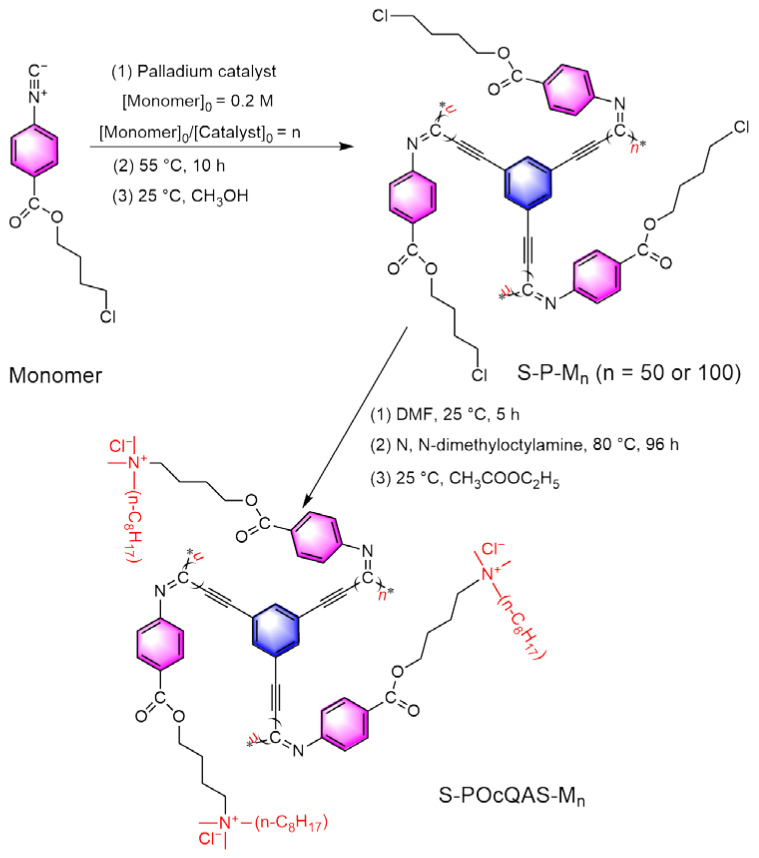
Chemical structure and synthetic procedures of S-POcQASs.

The S-POcQASs were synthesized by a process similar to L-POcQASs that was described in our previous work [28], except that the linear palladium catalyst was replaced by a star-shaped palladium catalyst. The synthesis route of targeted products is shown in Scheme 3.

### 2.5. Antimicrobial Activity Test of S-POcQASs

The antimicrobial activity was evaluated by determining the minimum inhibitory concentrations (MICs) of the S-POcQASs against *S. aureus* and *E. coli* using the TTC colorimetric method. The inoculated and incubated *E. coli* and *S. aureus* cells were diluted to 10^6^~10^7^ CFU/mL with LB broth. Then, 100 µL of bacteria was incubated in each well of a 96-well plate and cultured in a 100 μL growth medium. The S-POcQASs were diluted with sterile water to a concentration of 20 mg/mL, and it was added to the 96-well plate and adjusted to the required concentration (2.5~5000 μg/mL). The negative control group consisted of the nutritional broth, and the positive control sample was made up without S-POcQASs. After that, the agar plates were incubated in an incubator at 37 °C for 24 h. Finally, the antibacterial activity evaluation was carried out three times under the same experimental conditions.

### 2.6. Characterizations

^1^H NMR spectra and ^13^C NMR spectra of the samples were recorded by a Bruker Avance III-500 (Bruker Co., Ltd., Fällanden, Switzerland) using DMSO-d6 and CDCl_3_ as the solvent. The chemical structures of the samples were verified by Fourier transform infrared (FTIR) spectra (Spectrum 100, PerkinElmer, Waltham, MA, USA).

## 3. Results

### 3.1. Characterization of Polymers and Compounds

The structures of the polymers and compounds were verified by ^1^H NMR spectroscopy, ^13^C NMR spectroscopy and gel permeation chromatography (GPC). The number-averaged molecular masses (*M_n_*) and polydispersity indices (PDI = *M_w_*/*M_n_*) of these polymers are determined from GPC analysis and listed in Table 1. It was found that the molecular weight of these polyisocyanides increases gradually as the ratio of [Monomer]_0_/[Catalyst]_0_ increased from 50 to 150. In addition, all polymers exhibited a relatively high molecular weight and a relatively low polydispersity index (PDI ≈ 1.1). These results indicate that the polyisocyanides with different molecular weights can be polymerized in a controlled manner by initiating the polymerization of isocyanide monomers through palladium catalysts. It is worth noting that the molecular weight of S-P-M_n_ is nearly three times that of L-P-M_n_ when the [Monomer]_0_/[Catalyst]_0_ is the same, which can be ascribed to construction of a palladium catalyst with a star-shaped structure.

**Figure 1:**
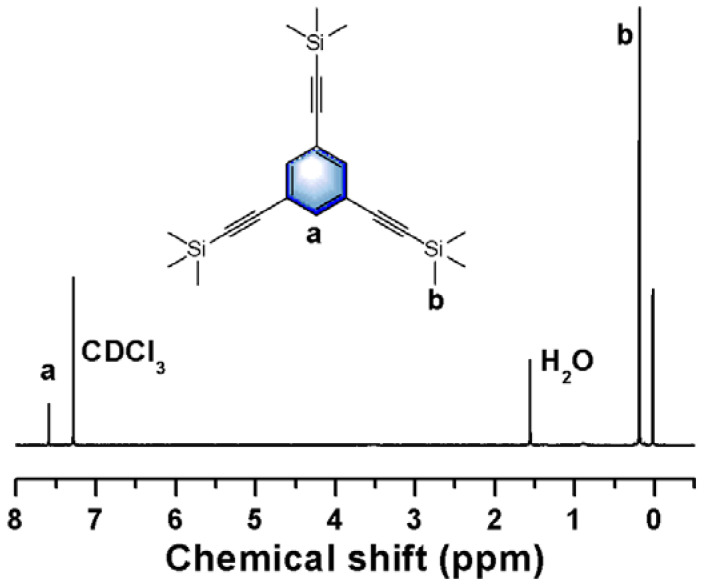
^1^H NMR spectrum of intermediate **a**.

Based on the above analysis, the catalyst plays a crucial role in the controllable preparation of polymers. Therefore, ^1^H NMR and ^13^C NMR were performed to confirm the structure of intermediates and the palladium catalyst. The ^1^H NMR spectrum of intermediate **a** is shown in Figure 1. The signal peak at δ = 7.59 ppm corresponded to unsubstituted hydrogen protons on the benzene ring, while the peak at δ = 0.19 ppm corresponded to hydrogen protons of –Si(CH_3_)_3_ groups, confirming the successful synthesis of intermediate **a**.

**Figure 2:**
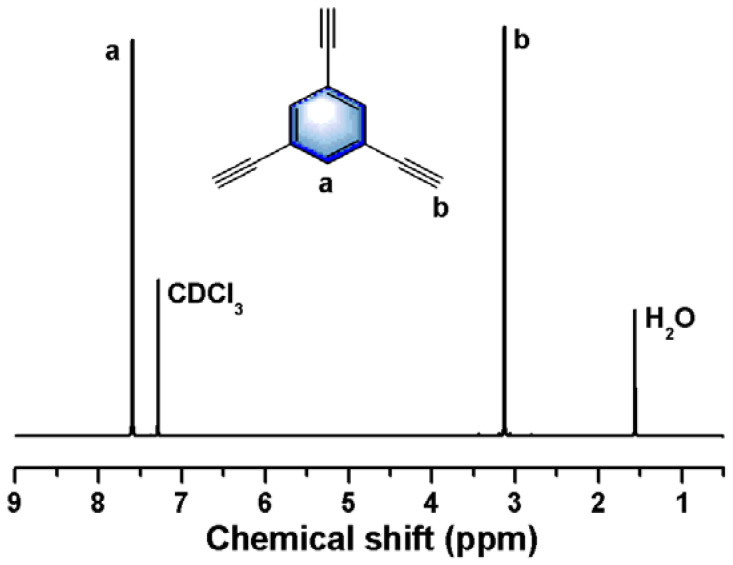
^1^H NMR spectrum of intermediate **b**.

Figure 2 showed the ^1^H NMR spectrum of intermediate **b**. The signal at δ = 7.66 ppm was attributed to unsubstituted hydrogen protons on the benzene ring, while the peak at δ = 3.13 ppm was assigned to hydrogen protons of –C≡C–H groups, which confirmed that intermediate **b** was synthesized successfully.

**Figure 3:**
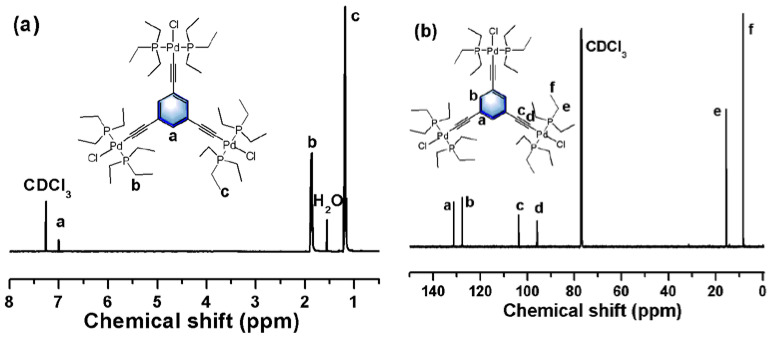
(**a**) ^1^H NMR and (**b**) ^13^C NMR spectra of palladium catalyst.

Figure 3 shows the ^1^H NMR and ^13^C NMR spectra of the palladium catalyst. As shown in Figure 3a, the signal at δ = 6.98 ppm was attributed to hydrogen protons on the benzene ring, while characteristic peaks at 1.84–1.95 and 1.10–1.16 ppm were attributed to the protons of –CH_2_– and –CH_3_ of –PEt_3_, respectively, indicating the successful synthesis of the palladium catalyst. The structure and component of the palladium catalyst was further verified by the ^13^C NMR spectrum (Figure 3b). The peaks at δ = 130.79 ppm and δ = 128.61 ppm were the characteristic peaks of unsubstituted carbon atoms and carbon atoms directly connected to the alkyne group in the benzene ring block, respectively. The peak at δ = 104.22 ppm represented the carbon atom peak of the alkyne group near the benzene ring. The signal at δ = 96.64 ppm was attributed to carbon atoms coordinated with palladium on the alkyne group. The peaks at δ = 15.44 ppm and δ = 8.33 ppm are the characteristic peaks of carbon atoms of –CH_2_– and –CH_3_ of –PEt_3_, respectively. These results demonstrate that the palladium catalyst with a star-shaped structure was constructed by introducing –Pd(PEt_3_)_2_Cl groups into intermediate **b**.

**Figure 4:**
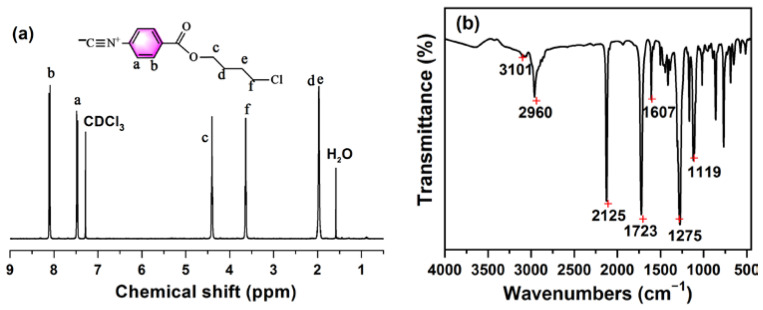
(**a**) ^1^H NMR and (**b**) FT-IR spectra of isonitrile monomer (The + in the graph represents the peak position).

Figure 4 shows the ^1^H NMR and FT-IR spectra of the isonitrile monomer. As shown in Figure 4a, δ = 8.06–8.08 ppm are the peaks of hydrogen protons on the benzene ring near the ester group, while δ = 7.44–7.46 ppm are the peaks of the other hydrogen protons on the benzene ring. Meanwhile, δ = 4.34–4.36 ppm at position c are the peaks of hydrogen protons in the methylene group connected to the ester group, δ = 3.59–3.62 ppm at position f are the peaks of hydrogen protons in the methylene group connected to the chlorine atom, and δ = 1.94–1.96 ppm at position d and position e are the peaks of the other hydrogen protons on 4-chloro-1-butyl group. In addition, FT-IR spectrometry was used to further characterize the structure of isonitrile monomer (Figure 4b). The band at 2125 cm^−1^ represents the C≡N stretching vibration. Furthermore, Figure 4b exhibited the characteristic bands corresponding to C=O stretching (1723 cm^−1^), C=C aromatic stretching (1607 cm^−1^), methylene C-H bending (1275 cm^−1^), and ester group C-O stretching (1119 cm^−1^). These results showed that the isonitrile monomer had been successfully obtained.

**Figure 5:**
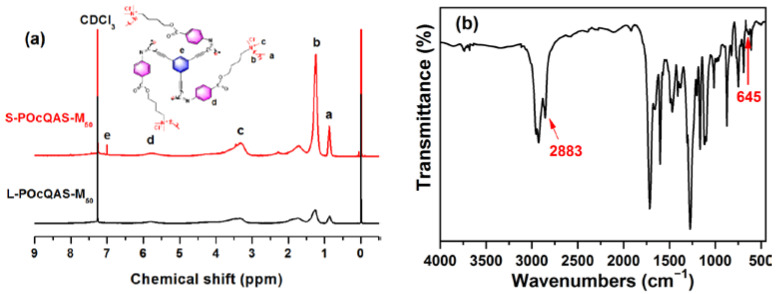
(**a**) ^1^H NMR and (**b**) FT-IR spectra of S-POcQAS-M_50._

In our previous work, L-POcQAS-M_n_ was successfully obtained by a simple quaternization reaction using N, N-dimethyloctylamine as the alkyl halides [28]. In this work, the S-POcQAS-M_n_ was synthesized by a process that was similar to L-POcQASs, except that the linear palladium catalyst was replaced by a star-shaped palladium catalyst. The molecular structure of the S-POcQAS-M_50_ sample was investigated by ^1^H NMR and FT-IR spectra. As shown in Figure 5a, the broad signal at δ = 3.16 ppm was attributed to hydrogen protons of –N^+^(CH_3_)_2_–, while characteristic peaks at 1.26 and 0.86 ppm were attributed to the protons of –CH_2_– and –CH_3_ of –(CH_2_)_7_CH_3_, respectively. Similar signal peaks could be obtained in the L-POcQAS-M_50_ sample, indicating that the S-POcQAS-M_50_ had been successfully obtained. In the FT-IR spectrum presented in Figure 5b, the absorption band located at 2883 cm^−1^ originated from the C-H stretching vibration in the introduced quaternary ammonium groups. Furthermore, the absorption peak of C-Cl stretching vibration at 645 cm^−1^ can hardly be observed, indicating that the quaternization of polyisocyanine was successfully achieved by using N, N-dimethyloctylamine, which matches well with the ^1^H NMR results.

### 3.2. Solubility and Antibacterial Properties

Since the antimicrobial properties of antimicrobial agents are mainly studied in aqueous solutions, the antimicrobial activity of QAS is suggested to be more related to its solubility. It is well known that polyisocyanide is insoluble in water. However, its solubility can be changed by modification of its side chain using tertiary amines. Our previous study found that L-POcQASs with n-octyl side chains had the best water solubility, suggesting that the optimal length of the alkyl side chain was 8. In this work, we also found that both S-POcQAS-M_50_ and S-POcQAS-M_100_ with n-octyl side chains exhibited high water solubility (Table 2). On the one hand, S-POcQAS has a branched structure, and on the other hand, the star-shaped structure of S-POcQAS increases the density of hydrophilic cations.

**Figure 6:**
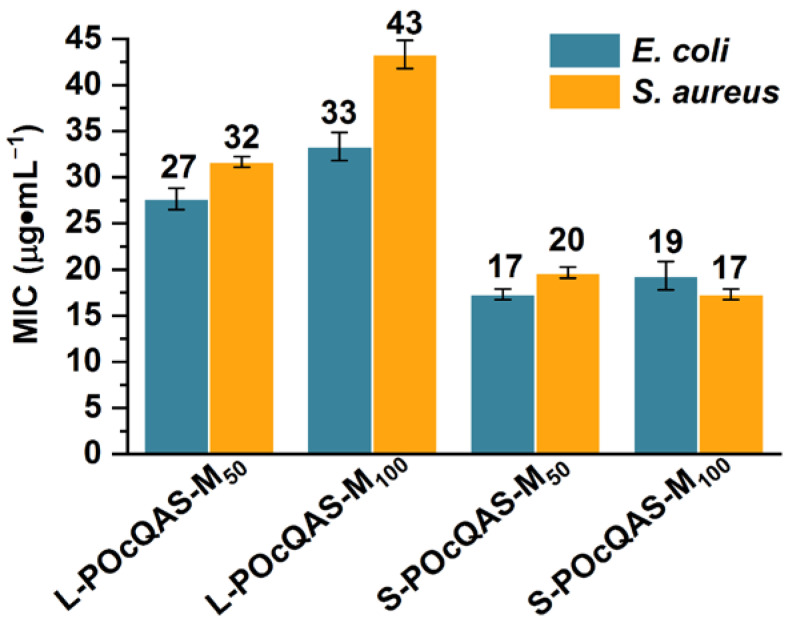
Minimum inhibitory concentrations (MICs) (μg/mL) of synthesized L-POcQASs and S-POcQASs against *S. aureus* and *E. coli*.

The antibacterial performance of the L-POcQAS-M_50_, L-POcQAS-M_100_, S-POcQAS-M_50_, and S-POcQAS-M_100_ were evaluated by using the minimum inhibitory concentrations (MICs) method. As shown in Figure 6, L-POcQASs demonstrated an apparent antibacterial effect, but it was obviously lower than that of S-POcQASs. Both S-POcQAS-M_50_ and S-POcQAS-M_100_ exhibited excellent antibacterial activity with MICs of 17 µg/mL and 19 μg/mL against *E. coli*, and 20 µg/mL and 17 μg/mL against *S. aureus*, respectively. However, the MICs of L-POcQAS-M_50_ and L-POcQAS-M_100_ were 27 µg/mL and 33 µg/mL on *E. coli*, and 32 µg/mL and 43 µg/mL on *S. aureus*, respectively. Remarkably, the improvement in antibacterial activity may be ascribed to their structural differences. It is well known that the sterilization mechanism of polymerized QAS antimicrobial materials is mostly contact-killing antibacterial. In other words, when the water-soluble QASs come into contact with the bacterial solution, the alkyl side chains of QASs can puncture the cell wall of bacteria, and then, the QAS groups with positive charge can be adsorbed onto the bacterial cell membrane with negative charge to cause the destruction of the cell membrane and the leakage of the inner cell contents, which will lead to the death of the bacteria. Generally, polymers with a branched structure have better water solubility than linear polymers. Furthermore, the unique star-shaped structure will increase the density of hydrophilic cations of POcQASs compared to the linear structure. Thus, as a result of the aforementioned factors, S-POcQASs exhibited approximately 1.5 times higher antibacterial activity than those of L-POcQASs. This work provides an effective and promising method for designing and preparing QAS antimicrobial materials.

## 4. Conclusions

In summary, two water-soluble antibacterials, L-POcQASs and S-POcQASs, have been synthesized by initiating the polymerization of isonitrile monomers with linear and star-shaped palladium catalysts, respectively, which is followed by quaternization by N, N-dimethyloctylamine. All the ^1^H NMR, FT-IR, and GPC results indicated that S-POcQASs were synthesized successfully. In addition, the synthesized S-POcQASs have high water solubility, and they exhibited higher antibacterial activities than L-POcQASs. Compared with L-POcQASs, S-POcQASs with hyperbranched structure possess more peripheral alkyl chains, more terminal cations and higher positive charge density, which facilitates the attraction and lysis of bacteria. Notably, the MIC of S-POcQAS-M_50_ to Gram-negative *E. coli* and Gram-positive *S. aureus* was as low as 17 µg/mL and 20 µg/mL, respectively, which suggests that S-POcQAS-M_50_ is a potential candidate as a kind of broad-spectrum antimicrobial agent.

## Figures and Tables

**Table 1:** Polymerization results of PQASs with palladium catalysts as initiator at 55 °C.

Entry	M/C *^a^*	Solvent	Polymer	*M*_n_ (g/mol) *^b^*	*M*_w_/*M*_n_*^c^*	Yield
1	50	THF	L-P-M_50_	10,125	1.12	88.2%
2	100	THF	L-P-M_100_	21,310	1.09	92.1%
3	150	THF	L-P-M_150_	30,051	1.13	79.4%
4	50	THF	S-P-M_50_	29,775	1.16	88.4%
5	100	THF	S-P-M_100_	59,611	1.11	94.6%

*^a^* M/C = monomer to palladium catalyst. *^b^* Determined by gel permeation chromatography (GPC) relative to polystyrene standards. *^c^* Polydispersity index calculated by GPC.

**Table 2:** Water solubility of L-POcQASs and S-POcQASs.

Entry	1	2	3	4
Compound	L-POcQAS-M_50_	L-POcQAS-M_100_	S-POcQAS-M_50_	S-POcQAS-M_100_
Solubility	14 mg/mL	11 mg/mL	13 mg/mL	9 mg/mL

## Data Availability

Not applicable.

## References

[1] Gao J., Li L., Duan L., Yang M., Zhou X., Zheng Q., Ou Y., Li Z., Lai F.Y. (2022). Exploring antibiotic consumption between urban and sub-urban catchments using both parent drugs and related metabolites in wastewater-based epidemiology. Sci. Total Environ..

[2] Durand-Reville T.F., Miller A.A., O’Donnell J.P., Wu X., Sylvester M.A., Guler S., Iyer R., Shapiro A.B., Carter N.M., Velez-Vega C. (2021). Rational design of a new antibiotic class for drug-resistant infections. Nature.

[3] Wu S., Hulme J.P. (2021). Recent advances in the detection of antibiotic and multi-drug resistant salmonella: An update. Int. J. Mol. Sci..

[4] Haitao Y., Yifan C., Mingchao S., Shuaijuan H. (2021). A novel polymeric nanohybrid antimicrobial engineered by antimicrobial peptide MccJ25 and chitosan nanoparticles exerts strong antibacterial and anti-inflammatory activities. Front. Immunol..

[5] Bueno J., Virto L., Toledano-Osorio M., Figuero E., Toledano M., Medina-Castillo A.L., Osorio R., Sanz M., Herrera D. (2022). Antibacterial effect of functionalized polymeric nanoparticles on titanium surfaces using an in vitro subgingival biofilm model. Polymers.

[6] Wu S., Xu J., Zou L., Luo S., Yao R., Zheng B., Liang G., Wu D., Li Y. (2021). Long-lasting renewable antibacterial porous polymeric coatings enable titanium biomaterials to prevent and treat peri-implant infection. Nat. Commun..

[7] Chen X., Chen Y., Lv S., Zhang L., Ye R., Ge C., Huang D., Zhang S., Cai Z. (2021). New type of borneol-based fluorine-free superhydrophobic antibacterial polymeric coating. Des. Monomers. Polym..

[8] Barros C.H.N., Hiebner D.W., Fulaz S., Vitale S., Quinn L., Casey E. (2021). Synthesis and self-assembly of curcumin-modified amphiphilic polymeric micelles with antibacterial activity. J. Nanobiotechnol..

[9] Olmos D., Gonzalez-Benito J. (2021). Polymeric materials with antibacterial activity: A Review. Polymers.

[10] Luo H., Yin X.Q., Tan P.F., Gu Z.P., Liu Z.M., Tan L. (2021). Polymeric antibacterial materials: Design, platforms and applications. J. Mater. Chem. B.

[11] Durak S., Arasoglu T., Ates S.C., Derman S. (2020). Enhanced antibacterial and antiparasitic activity of multifunctional polymeric nanoparticles. Nanotechnology.

[12] Padnya P.L., Terenteva O.S., Akhmedov A.A., Iksanova A.G., Shtyrlin N.V., Nikitina E.V., Krylova E.S., Shtyrlin Y.G., Stoikov I.I. (2021). Thiacalixarene based quaternary ammonium salts as promising antibacterial agents. Bioorg. Med. Chem..

[13] Lee C.Y., Chen Y.T., Lee B.S., Chang C.C. (2019). Suppressing antibacterial resistance: Chemical binding of monolayer quaternary ammonium salts to polymethyl methacrylate in an aqueous solution and its clinical efficacy. Int. J. Mol. Sci..

[14] Zhang Y., He X., Ding M., He W., Li J., Li J., Tan H. (2018). Antibacterial and biocompatible cross-linked waterborne polyurethanes containing gemini quaternary ammonium salts. Biomacromolecules.

[15] Ermolaev V.V., Arkhipova D.M., Miluykov V.A., Lyubina A.P., Amerhanova S.K., Kulik N.V., Voloshina A.D., Ananikov V.P. (2021). Sterically hindered quaternary phosphonium salts (QPSs): Antimicrobial activity and hemolytic and cytotoxic properties. Int. J. Mol. Sci..

[16] Chen Y., Tan W., Li Q., Dong F., Gu G., Guo Z. (2018). Synthesis of inulin derivatives with quaternary phosphonium salts and their antifungal activity. Int. J. Biol. Macromol..

[17] Gu G., Erisen D.E., Yang K., Zhang B., Shen M., Zou J., Qi X., Chen S., Xu X. (2022). Antibacterial and anti-inflammatory activities of chitosan/copper complex coating on medical catheters: In vitro and in vivo. J. Biomed. Mater. Res. B Appl. Biomater..

[18] Yuan H., Li W., Song C., Huang R. (2022). An injectable supramolecular nanofiber-reinforced chitosan hydrogel with antibacterial and anti-inflammatory properties as potential carriers for drug delivery. Int. J. Biol. Macromol..

[19] Yu D., Feng J., You H., Zhou S., Bai Y., He J., Cao H., Che Q., Guo J., Su Z. (2022). The microstructure, antibacterial and antitumor activities of chitosan oligosaccharides and derivatives. Mar. Drugs.

[20] Khayrova A., Lopatin S., Shagdarova B., Sinitsyna O., Sinitsyn A., Varlamov V. (2022). Evaluation of antibacterial and antifungal properties of low molecular weight chitosan extracted from hermetia illucens relative to crab chitosan. Molecules.

[21] Zhang X., Han D., Pei P., Hao J., Lu Y., Wan P., Peng X., Lv W., Xiong W., Zeng Z. (2019). In vitro antibacterial activity of isopropoxy benzene guanidine against multidrug-resistant enterococci. Infect. Drug Resist..

[22] Kliewer S., Wicha S.G., Broker A., Naundorf T., Catmadim T., Oellingrath E.K., Rohnke M., Streit W.R., Vollstedt C., Kipphardt H. (2020). Contact-active antibacterial polyethylene foils via atmospheric air plasma induced polymerisation of quaternary ammonium salts. Colloids Surf. B Biointerfaces.

[23] Liu W.S., Wang C.H., Sun J.F., Hou G.G., Wang Y.P., Qu R.J. (2015). Synthesis, characterization and antibacterial properties of dihydroxy quaternary ammonium salts with long chain alkyl bromides. Chem. Biol. Drug Des..

[24] Wang C.H., Xie X.R., Liu W.S., Hou G.G., Sun J.F., Zhao F., Cong W., Li H.J., Xin W.Y. (2017). Quaternary ammonium salts substituted by 5-phenyl-1,3,4-oxadiazole-2-thiol as novel antibacterial agents with low cytotoxicity. Chem. Biol. Drug Des..

[25] Zhang L., Feng X.Z., Xiao Z.Q., Fan G.R., Chen S.X., Liao S.L., Luo H., Wang Z.D. (2021). Design, synthesis, antibacterial, antifungal and anticancer evaluations of novel beta-pinene quaternary ammonium salts. Int. J. Mol. Sci..

[26] Dong Y., Liu L., Sun J., Peng W., Dong X., Gu Y., Ma Z., Gan D., Liu P. (2021). Phosphonate/quaternary ammonium copolymers as high-efficiency antibacterial coating for metallic substrates. J. Mater. Chem. B.

[27] Worley B.V., Slomberg D.L., Schoenfisch M.H. (2014). Nitric oxide-releasing quaternary ammonium-modified poly(amidoamine) dendrimers as dual action antibacterial agents. Bioconjugate Chem..

[28] Zhang H., Liu L., Hou P., Liu J., Fu S. (2021). Design, synthesis, antibacterial, and antitumor activity of linear polyisocyanide quaternary ammonium salts with different structures and chain lengths. Molecules.

[29] Meyers S.R., Juhn F.S., Griset A.P., Luman N.R., Grinstaff M.W. (2008). Anionic amphiphilic dendrimers as antibacterial agents. J. Am. Chem. Soc..

[30] Bakhshi H., Agarwal S. (2017). Hyperbranched polyesters as biodegradable and antibacterial additives. J. Mater. Chem. B.

[31] Asri L.A.T.W., Crismaru M., Roest S., Chen Y., Ivashenko O., Rudolf P., Tiller J.C., van der Mei H.C., Loontjens T.J.A., Busscher H.J. (2014). A shape-adaptive, antibacterial-coating of immobilized quaternary-ammonium compounds tethered on hyperbranched polyurea and its mechanism of action. Adv. Funct. Mater..

[32] Wang Y., Yuan X., Li H., Liu L., Zhao F., Wang G., Wang Q., Yu Q. (2020). Antibacterial and drug-release dual-function membranes of cross-linked hyperbranched cationic polymers. React. Funct. Polym..

[33] Chen C.Z., Cooper S.L. (2002). Interactions between dendrimer biocides and bacterial membranes. Biomaterials.

[34] Chen C.Z., Beck-Tan N.C., Dhurjati P., van Dyk T.K., LaRossa R.A., Cooper S.L. (2000). Quaternary ammonium functionalized poly (propylene imine) dendrimers as effective antimicrobials: Structure− activity studies. Biomacromolecules.

[35] Cuneo T., Gao H. (2020). Recent advances on synthesis and biomaterials applications of hyperbranched polymers. Wiley Interdiscip. Rev. Nanomed. Nanobiotechnol..

[36] Xue Y.X., Zhu Y.Y., Gao L.M., He X.Y., Liu N., Zhang W.Y., Yin J., Ding Y., Zhou H., Wu Z.Q. (2014). Air-stable (phenylbuta-1,3-diynyl)palladium(II) complexes: Highly active initiators for living polymerization of isocyanides. J. Am. Chem. Soc..

